# The role of miR-6884-5p in epithelial-mesenchymal transition in non-small cell lung cancer

**DOI:** 10.18632/aging.205474

**Published:** 2024-01-24

**Authors:** Lianyong Zhang, Wei Chi, Xue Wang, Jingjing Li, Fei Li, Yuxia Ma, Qianyun Zhang

**Affiliations:** 1 Department of Pulmonary and Critical Care Medicine (PCCM) Ward II, Cangzhou Central Hospital, Cangzhou 061000, Hebei, China; 2 Department of Geriatrics, Cangzhou Central Hospital, Cangzhou 061000, Hebei, China

**Keywords:** NSCLC, miR-6884-5p, S100A16, correlation, patients

## Abstract

Significant progress has been made in the management of non-small cell lung cancer (NSCLC), though a big barrier remains, which is epithelial–mesenchymal transition (EMT). Our study aimed to evaluate the function of miR-6884-5p and S100A16 in EMT-aggravated NSCLC. The tumor tissues and adjacent tissues from 92 NSCLC patients were collected to analyze the expression of miR-6884-5p and S100A16. Then lung cancer cell line A549 was co-transfected with miR-6884-5p mimics and S100A16 to further evaluate their function. Compared to adjacent tissues, low expression of miR-6884-5p was observed in the NSCLC tissues and associated with severe NSCLC progression. MiR-6884-5p expression was negatively correlated with EMT in NSCLC. Luciferase assay data revealed that miR-6884-5p could directly bind to the 3’UTR of S100A16 and inhibited the expression of S100A16 in A549 cells. Moreover, miR-6884-5p mimics significantly ameliorated EMT progression, and overexpression of S100A16 could reverse the inhibitory effect of miR-6884-5p in A549 cells. MiR-6884-5p inhibited EMT through directly targeting S100A16 in NSCLC. Our findings suggest that miR-6884-5p could be a diagnostic marker of NSCLC, as well as a potential candidate for NSCLC treatment.

## INTRODUCTION

Non-small cell lung cancer (NSCLC) is one subtype of lung cancer, and takes up 85% of lung cancer cases, with an estimated 500,000 new cases and 300,000 deaths each year. NSCLC accounts for about 80% of all lung cancers. The incidence of NSCLC rises as the median age at diagnosis falls. However, approximately 75% of patients who are diagnosed with NSCLC are in the middle and late stages with an extremely low 5-year survival rate. Despite advances in diagnostic and therapeutic techniques, the 5-year overall survival rate for NSCLC is still as low as 40%. Hence, evaluating the molecular mechanisms of NSCLC and discovering its new biomarkers are of great significance for the diagnosis and management of NSCLC [1, 2].

The S100 protein family contains more than 25 members, which are mostly involved in the cellular processes, such as motility, contraction, cell differentiation, cell growth, and cell cycle progression [3–5]. Most of them are detected not only in many human cancers, such as colorectal cancer, ovarian cancer gastric cancer, pancreatic cancer, and lung cancer [6–10], but also in multiple stages of cancer progression with a correlation of a poor prognosis through regulating cellular functions, including. cell migration, invasion, apoptosis and proliferation. Among them, S100A16 is the most studied one, which is highly expressed in several cancers, such as oral squamous cell carcinoma, breast cancer, colorectal cancer, prostate cancer, cervical carcinoma, and lung cancer [11–15]. Therefore, S100A16 is regarded as a key modulator for multiple cancers. Previous publication also clarified that S100A16 was an independent indicator of prognosis in NSCLC [16]. However, no more related research was continued. Thus, we aimed to explore the function of S100A16 in NSCLC.

It is widely acknowledged that micro-RNAs (miRNAs) are involved in the differentiation, proliferation and apoptosis of tumor cells, including NSCLC cells [17, 18]. There are some candidate miRNAs in NSCLC that can be used as oncogenic or tumor suppressor factors, and abnormal miRNA levels may serve as useful biomarkers for NSCLC diagnosis [19, 20]. MiR-6884-5p is an emerging miRNA that mainly acts in the progression of multiple cancers, such as ovarian cancer, gastric cancer, and esophageal squamous cell carcinoma [21–23]. MiR-6884-5p was reported to directly target S100A16 to regulate gastric cancer cells [23]. However, the role of miR-6884-5p in NSCLC is unknown. Herein, the aim of our study was to explore the role of miR-6884-5p/S100A16 axis in NSCLC.

## RESULTS

### MiR-6884-5p was negatively correlated with S100A16 in NSCLC tissue

**Figure 1:**
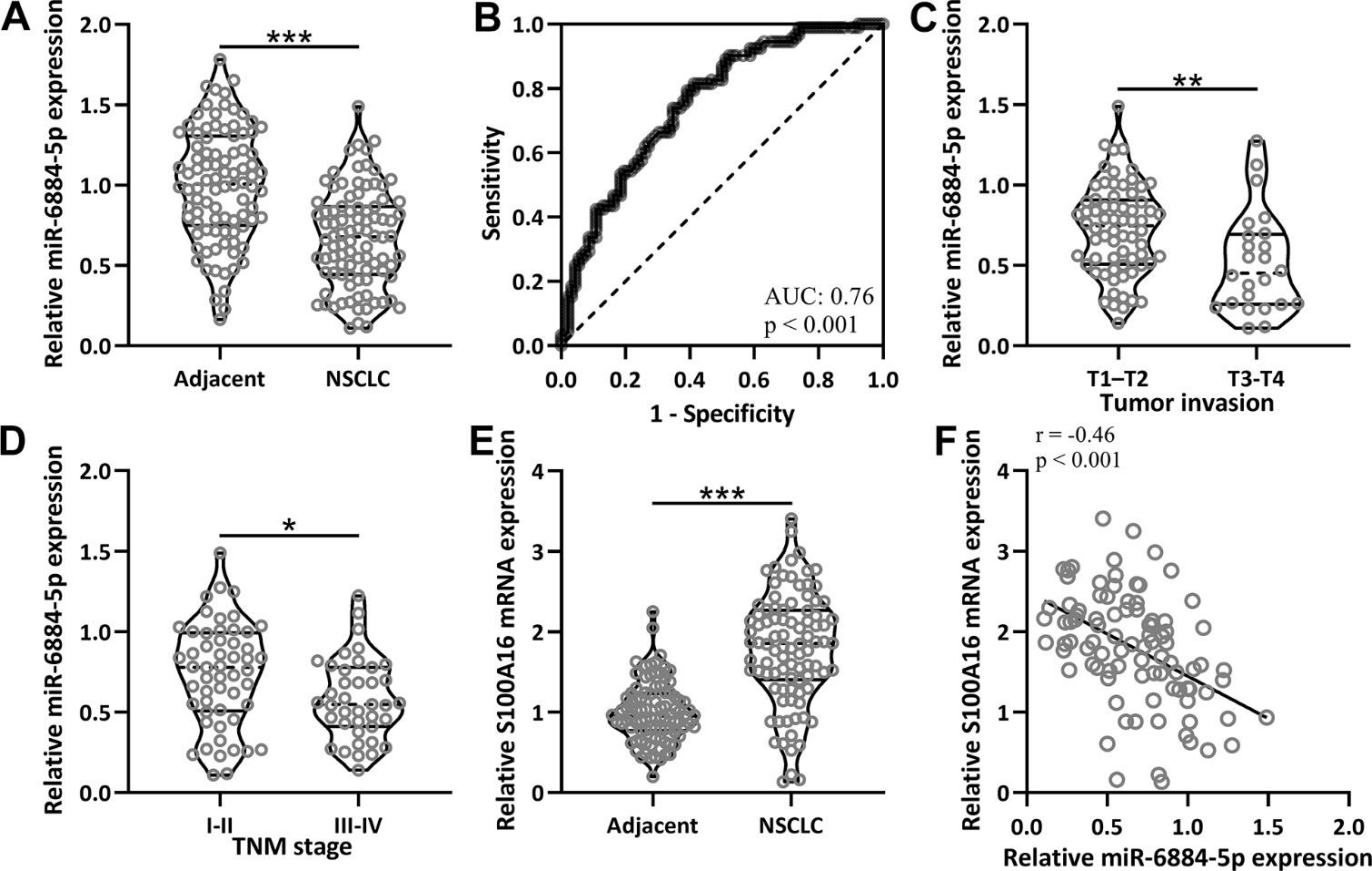
**The expressions of miR-6884-5p and S100A16 in non-small cell lung cancer tissues.** (**A**) qRT-PCR was used to determine the expressions of miR-6884-5p between adjacent tissues and NSCLC tissues (n = 92 for each). (**B**) ROC analysis of the expressions of miR-6884-5p for the diagnosis of NSCLC. (**C**) Comparison of the expressions of miR-6884-5p between tumor invasion of T1-T2 (n = 68) and T3-T4 (n = 24). (**D**) Comparison of the expressions of miR-6884-5p between TNM stage of I-II (n = 53) and III-IV (n = 39). (**E**) qRT-PCR was used to determine the mRNA expressions of S100A16 between adjacent tissues and NSCLC tissues (n = 92 for each). Violin plot was used to show the data. *p < 0.05, **p < 0.01, ***p < 0.001 from unpaired t-test with Welch’s correction. (**F**) Pearson correlation coefficient analysis was employed to analyze the correlations of the expressions of miR-6884-5p and the mRNA expressions of S100A16 in NSCLC tissues (n = 92).

First, we compared miR-6884-5p expression between adjacent tissues and NSCLC tissues from 92 patients, and miR-6884-5p expression was significantly lower in NSCLC tissues than in the adjacent tissues (Figure 1A). Receiver operating characteristic (ROC) analysis data revealed that miR-6884-5p could serve as a diagnostic marker of NSCLC with a 0.76 area under the curve (AUC) (Figure 1B). Based on tumor invasion, these 92 NSCLC tissues could be divided into 68 T1-T2 stages and 24 T3-T4 stages, and miR-6884-5p expression was also lower in the T3-T4 stages than in the T1-T2 stages (Figure 1C). Moreover, 92 NSCLC tissues were also divided into the I-II stages (n-53) and the III-IV stages (n=39) according to the TNM, and miR-6884-5p was decreased in III-IV stage in comparison with I-II stage (Figure 1D). To evaluate the relationship between miR-6884-5p and S100A16 in NSCLC, we measured the mRNA expression of S100A16 in adjacent tissues and NSCLC tissues. As expected, S100A16 expression was higher in NSCLC tissues than in adjacent tissues (Figure 1E), and miR-6884-5p expression was negatively correlated with S100A16 expression in NSCLC tissues (Figure 1F).

### Low expression of miR-6884-5p was associated with severe NSCLC progression

To further evaluate the role of miR-6884-5p in NSCLC, 92 MSCLC tissues were also divided into two groups: Low expression (n=46) and high expression (n=46), according to the relative median expression of miR-6884-5p in the tumor tissue. Then we compared some clinical information between these two groups and found that different expressions of miR-6884-5p were associated with tumor invasion and TNM stage (Table 1). Moreover, lower expression of miR-6884-5p was associated with more severe NSCLC progression, which means that miR-6884-5p expression was low in the more severe tumor tissue. Meanwhile, age, gender, and lymph node metastasis had no effect on the expressions of miR-6884-5p.

**Table 1:** miR-6884-5p expression and clinicopathological outcomes in non-small cell lung cancer (NSCLC) patients (n = 92).

**Outcomes**	**miR-6884-5p expression**		**p-value**
	**Low expression (n = 46)**	**High expression (n = 46)**	
Age (years)			
< 60	19 (20.7%)	25 (27.2%)	0.297
≥ 60	27 (29.3%)	21 (22.8%)	
Gender			
Male	26 (28.3%)	24 (26.1%)	0.834
Female	20 (21.7%)	22 (23.9%)	
Tumor invasion			
T1–T2	29 (31.5%)	39 (42.4%)	0.031
T3-T4	17 (18.5%)	7 (7.6%)	
Lymph node metastasis			
N0	27 (29.3%)	31 (33.7%)	0.517
N1-3	19 (20.7%)	15 (16.3%)	
TNM stage			
I-II	20 (21.7%)	33 (35.9%)	0.011
III-IV	26 (28.3%)	13 (14.1%)	

Fisher’s exact test, TNM, tumor-node-metastasis.

### MiR-6884-5p expression was negatively correlated with epithelial–mesenchymal transition (EMT) in NSCLC

**Figure 2:**
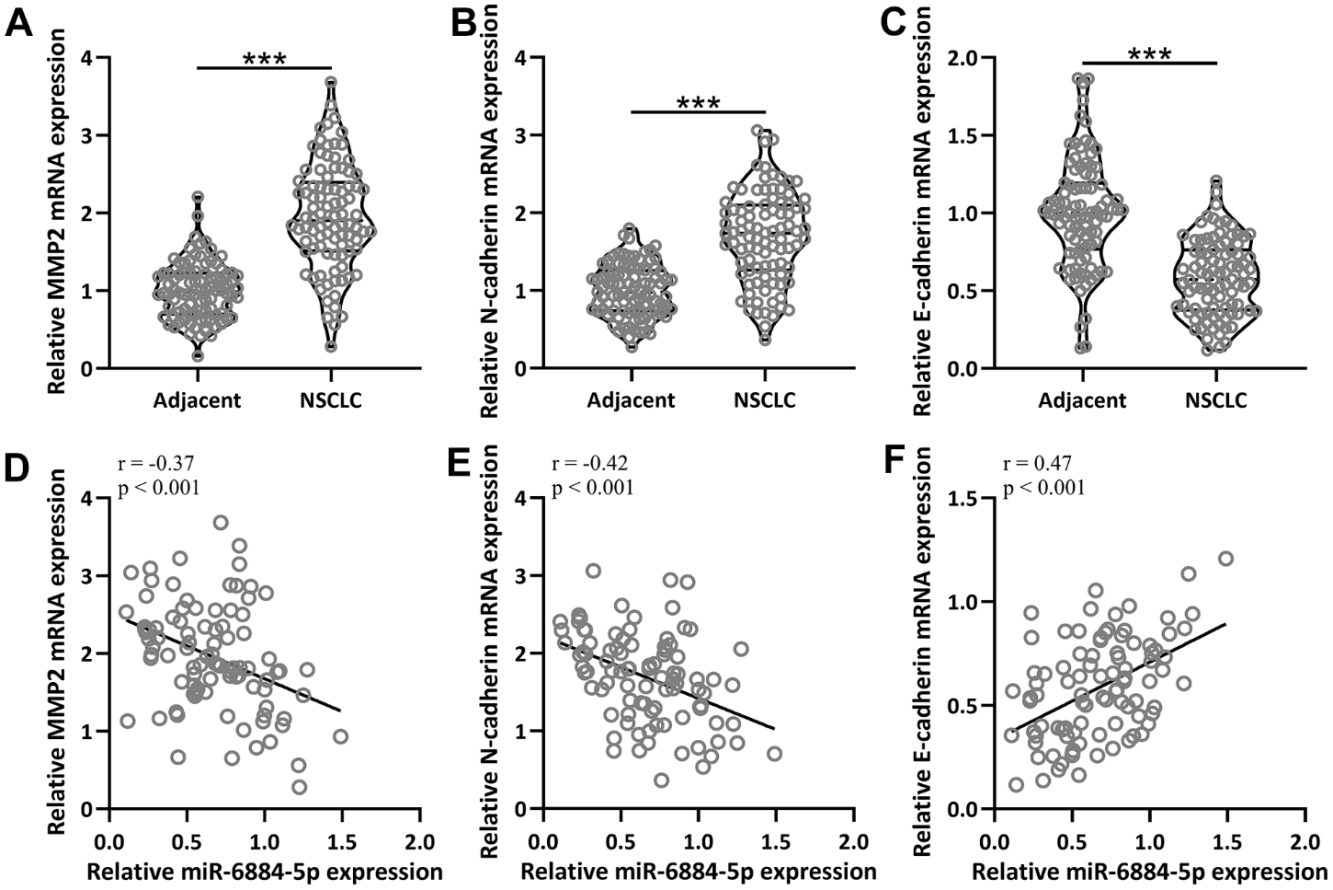
**The mRNA expressions of MMP2, N-cadherin and E-cadherin between adjacent tissues and NSCLC tissues (n = 92 for each).** (**A**–**C**) qRT-PCR was used to determine the mRNA levels of MMP2, N-cadherin and E-cadherin. Violin plot was used to show the data. *p < 0.05, **p < 0.01, ***p < 0.001 from unpaired t-test with Welch’s correction. (**D**–**F**) Pearson correlation coefficient analysis was employed to analyze the correlations of the expressions of miR-6884-5p and the mRNA expressions of MMP2, N-cadherin and E-cadherin in NSCLC tissues (n = 92).

EMT plays an important role in the progression of NSCLC. Therefore, we explored some important EMT-related genes both in the adjacent tissues and tumor tissues, including matrix metallopeptidase 2 (MMP2), N-cadherin, and E-cadherin [24, 25]. The expressions of MMP2 and N-cadherin were both higher in the NSCLC tissues than in the adjacent tissue (Figure 2A, 2B), and conversely, E-cadherin had a lower expression in the NSCLC tissue than in the adjacent tissues (Figure 2C). As expected, miR-6884-5p expression was negatively correlated with MMP2 (r=-0.37) and N-cadherin (r=-0.42) expression (Figure 2D, 2E), and positively correlated with E-cadherin (r=0.47) expression (Figure 2F). Thus, these data suggested that miR-6884-5p expression was negatively associated with EMT.

### S100A16 expression was positively correlated with EMT in NSCLC

**Figure 3:**
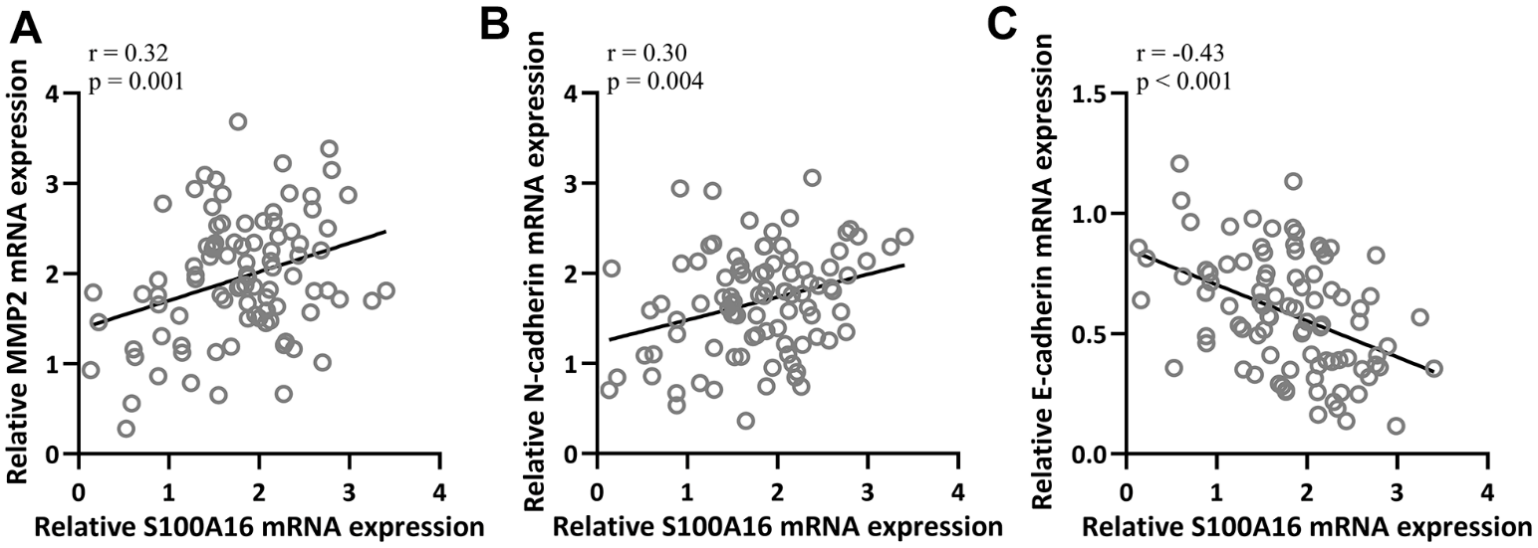
Pearson correlation coefficient analysis was employed to analyze the correlations of mRNA expressions of S100A16 and the mRNA expressions of MMP2 (**A**), N-cadherin (**B**) and E-cadherin (**C**) in NSCLC tissues (n = 92).

Next, we further evaluated the relationship between EMT and S100A16 in NSCLC. Pearson correlation coefficient analysis revealed that mRNA levels of S100A16 were positively correlated with MMP2 (r=0.32) and N-cadherin (r=0.30) respectively (Figure 3A, 3B), and negatively correlated with E-cadherin (r=-0.43) (Figure 3C) in NSCLC tissues. These data suggested that S100A16 expression was positively correlated with EMT in NSCLC.

### S100A16 expression and EMT were increased in NSCLC tissue

**Figure 4:**
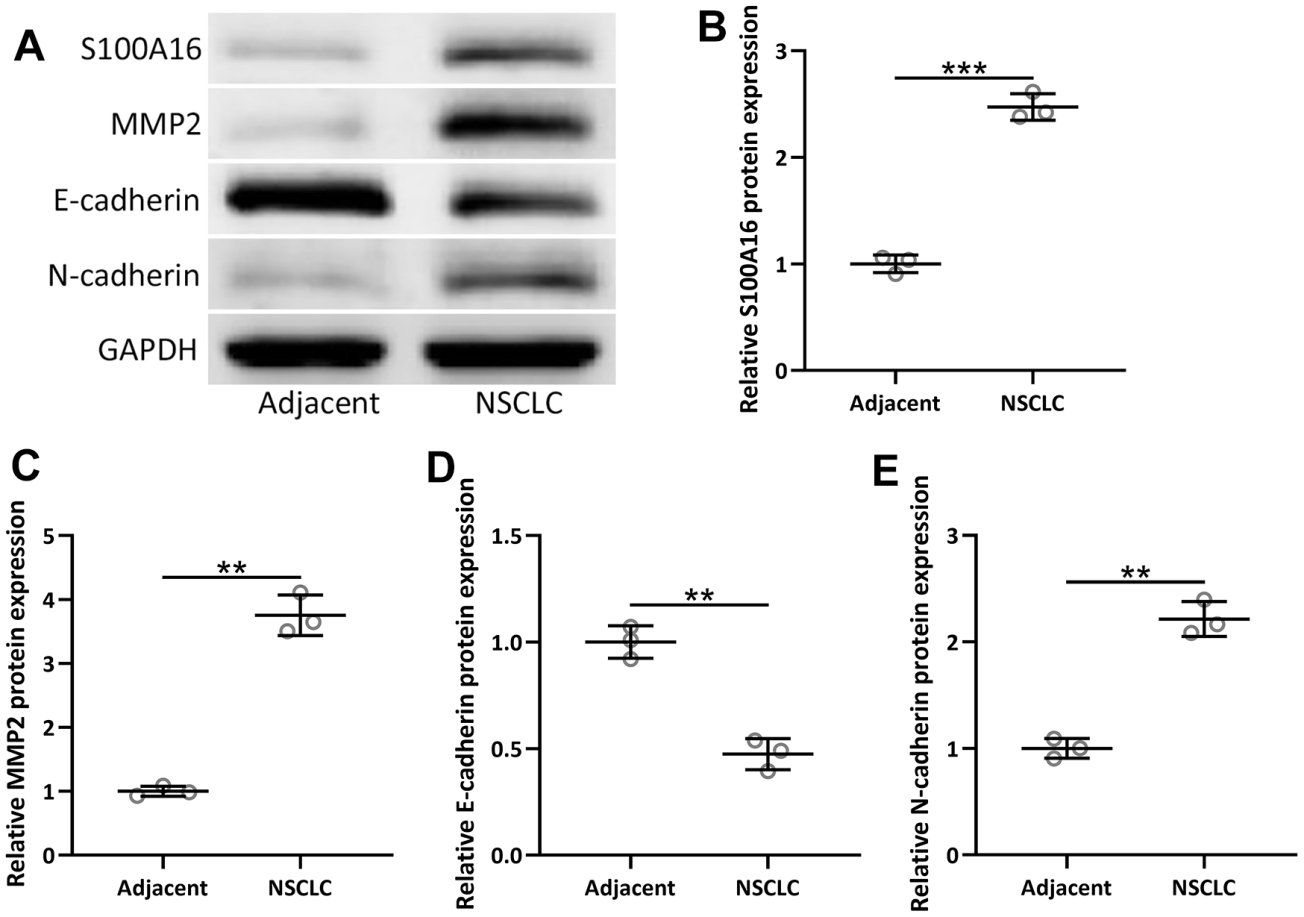
**The protein expressions of S100A16, MMP2, N-cadherin and E-cadherin between adjacent tissues and NSCLC tissues (n = 92 for each).** (**A**) Western blot was used to determine the protein expression. GAPDH was used as the loading control. The expressions were normalized to adjacent (**B**–**E**). Violin plot was used to show the data. *p < 0.05, **p < 0.01, ***p < 0.001 from unpaired t-test with Welch’s correction.

Besides mRNA level, the protein level of S100A16 and EMT-related proteins, including MMP2, N-cadherin, and E-cadherin, was also measured in 92 NSCLC patients. Compared to adjacent tissue, NSCLC tissues had increased protein levels of S100A16, MMP2, and N-cadherin with a decreased protein level of E-cadherin (Figure 4A–4F), which further confirmed that S100A16 and EMT were associated with NSCLC.

### MiR-6884-5p directly targeted S100A16 in NSCLC

**Figure 5:**
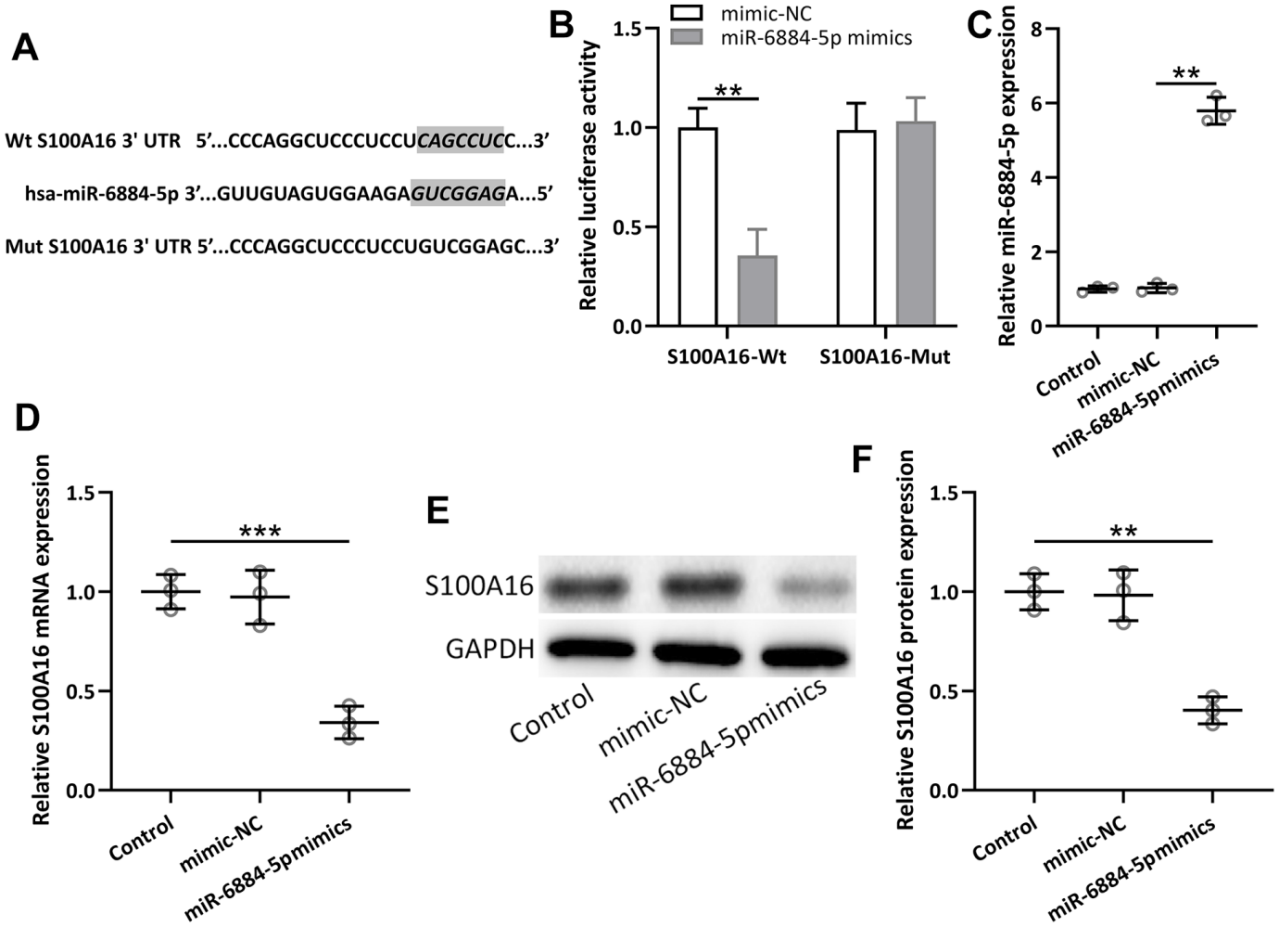
**miR-6884-5p targeted S100A16 and regulated the expression of S100A16 in A549 cells.** (**A**) The predicted binding sites of hsa-miR-6884-5p with wild-type 3′-UTR region of S100A16 mRNA are shown. A mutated 3′-UTR of S100A16 is also shown. (**B**) A549 cells were co-transfected with luciferase reporters containing Wt and/or mutant S100A16 3′-UTR with miR-6884-5p mimics and negative control. After 48 h of incubation, relative luciferase activities were measured. n = 3. Data were shown as means ± SD. **p < 0.01, Two-way ANOVA followed Turkey’s multiple comparisons test. C, A549 cells were transfected with miR-6884-5p mimics or negative control for 48 h. qRT-PCR was used to determine the expressions of miR-6884-5p (**C**) and the mRNA expressions of S100A16 (**D**). Western blot was used to determine the protein expressions of S100A16 (**E**). GAPDH was used as the loading control and the expressions were normalized to control (**F**). n = 3. Data were shown as Mean ± SD. **p < 0.01, ***p < 0.001 from Welch’s ANOVA test followed Dunnett’s T3 multiple comparisons test.

MiR-6884-5p was reported to directly target S100A16 in gastric cancer cells [23], thus miR-6884-5p was also expected to directly target S100A16 in NSCLC. The predicted binding sites of has-miR-6884-5p with 3’-UTR regions of S100A16 mRNA and a mutated 3’-UTR of S100A16 mRNA were shown in Figure 5A. Lung cancer cell line A549 cells were co-transfected with luciferase reporters containing wild-Type (WT) or mutated S100A16 3’-UTR and miR-6884-5p mimics/negative control. Luciferase data showed that miR-6884-5p directly binds to 3’-UTR of S100A16 mRNA through predicted binding sites, because the mutated one lost their binding activity (Figure 5B). Moreover, miR-6884-5p mimics significantly decreased both the mRNA level and protein level of S100A16 in A549 cells (Figure 5C–5F). Therefore, miR-6884-5p directly target the expression of S100A16 in lung cancer cells.

### MiR-6884-5p modulated EMT through S100A16 in lung cancer

**Figure 6:**
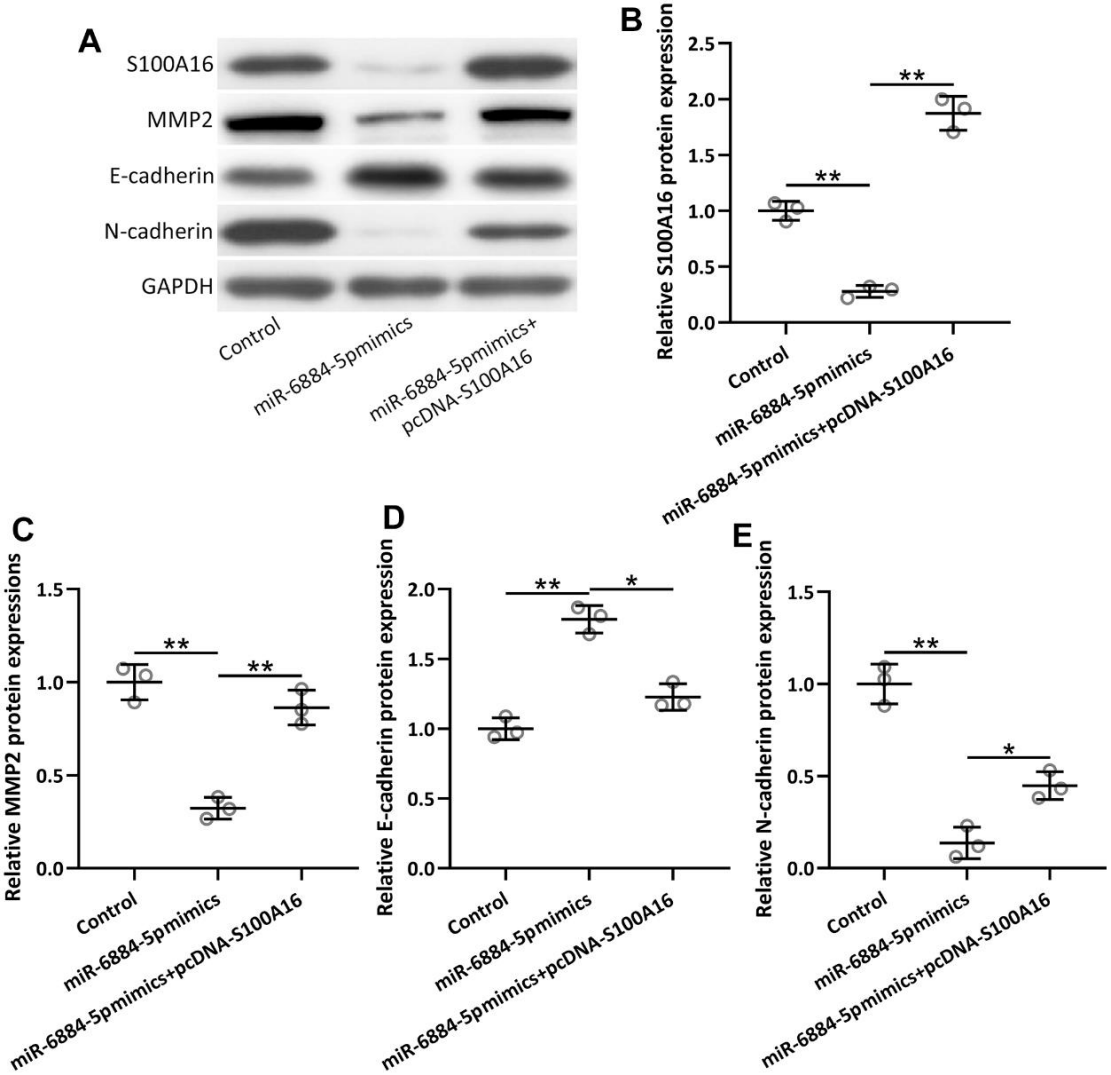
**A549 cells were transfected with miR-6884-5p mimics or miR-6884-5p mimics plus pcDNA-S100A16 for 48 h.** Western blotting was used to determine the protein expressions of S100A16, MMP2, N-cadherin and E-cadherin (**A**). GAPDH was used as the loading control and the expressions were normalized to control (**B**–**E**). n = 3. Data were shown as Mean ± SD. *p < 0.05, **p < 0.01 from Welch’s ANOVA test followed Dunnett’s T3 multiple comparisons test.

Furthermore, miR-6884-5p mimics significantly inhibited EMT progression in lung cancer, as reflected by increased protein levels of MMP2 and E-cadherin, and a decreased protein level of N-cadherin (Figure 6A–6E). Overexpression of S100A16 by pcDNA-S100A16 in A549 cells reversed the effect of miR-6884-5p mimics, which meant S100A16 overexpression could promote EMT in lung cancer cells even after transfection of miR-6884-5p mimics in A549 cells. Hence, these observations indicated that miR-6884-5p modulated EMT at least partially through S100A16 in NSCLC.

## DISCUSSION

Emerging evidence demonstrated the important roles of S100 proteins in tumorigenesis as well as cancer metastasis [26, 27], and S100 proteins have been recognized as critical modulators of cell growth, cell cycle progression, cell migration and invasion [3, 4, 28]. Moreover, various expressions of S100 proteins have been reported to be tightly associated with different human cancers, particularly lung cancer [6]. For example, S100A2 was detected to be highly expressed in NSCLC [29]. The expressions of S100A6 were different in different subtypes of lung cancer [30]. In recent years, more and more studies have focused on the role of S100A16 in tumorigenesis and cancer metastasis. S100A16 is considered an oncogene and a prognostic marker of multiple cancers, including pancreatic cancer, colorectal cancer [9, 31, 32], especially lung adenocarcinomas and small-cell lung cancer [15, 33, 34]. However, the role of S100A16 in NSCLC, which is one of the most aggressive subtypes of lung cancer, remains unknown. Therefore, based on the crucial function of S100A16 in some subtypes of lung cancer, our study aimed to explore the function of S100A16 in NSCLC.

Previous studies have shown that miRNAs are involved in the differentiation, proliferation, and apoptosis of tumor cells, including NSCLC cells [17, 18]. There are some candidate miRNAs in NSCLC that can be used as oncogenic or tumor suppressor factors, and abnormal miRNA levels may serve as useful biomarkers for NSCLC diagnosis [19, 20]. For example, miR-7 regulates apoptosis and malignant behavior of HeLa and C33A cells by targeting XIAP in lung cancer [35, 36]. Moreover, upregulating miR-145 is associated with the aggressive progression and poor prognosis of NSCLC patients [37]. Furthermore, miR-6884 was also an important regulator in multiple cancers. Long non-coding RNA (lncRNA) RP11-295G20.2 could bind to miR-6884-3p as a ceRNA to regulate hepatocellular carcinoma progression through Cyclin B1 (CCNB1) pathway [38]. Moreover, miR-6884-5p directly targeted S100A16 to regulate gastric cancer [23], which gave us a hint that miR-6884-5p might also target S100A16 to modulate NSCLC, based on the key role of S100A16 in lung-related cancer. And our study confirmed the regulatory role of miR-6884-5p in NSCLC by directly targeting S100A16.

However, besides S100A16, miR-6884-5p was reported to regulate l lncRNA LINC01224 as competitive endogenous RNA (ceRNA), and miR-6884–5p/Dishevelled segment polarity protein 3 (DVL3) axis also activated Wnt/β-catenin signaling pathway in squamous cell carcinoma [22]. This is the limitation of our current study that we did not determine whether LINC01224, DVL3, and Wnt/β-catenin signaling were involved in the progressions of NSCLC/ and whether miR-6884-5p could regulate them.

EMT drives metastasis during multiple cancer progressions, which plays a key role in cancer metastasis. For example, a previous publication revealed that EMT was associated with T cell infiltration during NSCLC [39]. More and more evidence indicated the progression of EMT aggravated NSCLC [40–43]. The management of NSCLC, which takes up 85% of lung cancer cases, has been had made big progress in the recent years [44]. However, the progression of EMT is still a barrier of NSCLC management [44]. Our study confirmed that EMT was negatively correlated with NSCLC by analyzing the expressions of typical markers of EMT between NSCLC tissues and adjacent tissues, including MMP2, N-cadherin, and E-cadherin. Therefore, our data suggested that inhibiting the progression of EMT could ameliorate NSCLC, which was consistent with previous publications. For example, a new Rho-associated protein kinase 1 inhibitor, Neferine suppressed the progression of EMT, leading to the management of NSCLC [45]. Furthermore, the RNA-binding protein quaking homolog 6 also suppressed NSCLC through inhibiting EMT [46].

Our study observed that overexpression of miR-6884-5p by transfecting miR-6884-5p mimics in A549 cells could significantly block the progression of EMT, as reflected by the decreased expressions of MMP2 and N-cadherin as well as increased expression of E-cadherin. Moreover, miR-6884-5p mimics also attenuated the expression of S100A16 in A549 cells, conversely overexpression of S100A16 in A549 cells, could reverse the inhibitory effect of miR-6884-5p on the expressions of MMP2, N-cadherin, and E-cadherin, which suggested that miR-6884-5p regulated EMT progression through S100A16, leading to the management of NSCLC.

There are some shortcomings should be noted. First, the current findings are based on the patients’ samples and A549 lung cancer cells, and lack the verification in the lung cancer model. We might further use a mouse model of lung cancer and wound healing to mimic EMT progression to explore the role of miR-6884-5p and S100A16 in NSCLC. Second, the detailed molecular mechanisms underlying the regulation of EMT by miR-6884-5p should be further explored.

## CONCLUSIONS

Our results demonstrate that miR-6884-5p is negatively correlated with S100A16 in NSCLC tissues, and low expression of miR-6884-5p is associated with severe NSCLC progression. In addition, miR-6884-5p expression is negatively correlated with EMT, while S100A16 expression is positively correlated with EMT in NSCLC. Furthermore, it is found that miR-6884-5p directly targets S100A16 and modulates EMT at least partially through S100A16 in NSCLC. The current study suggests that miR-6884-5p could be a diagnostic marker of NSCLC as well as the potential candidate approach for NSCLC treatment.

## MATERIALS AND METHODS

### Patients

The subjects of the study were 92 patients with NSCLC in Cangzhou Central Hospital who underwent surgical resection of lung cancer tissue. Adjacent tissues were collected for comparison. All patients were confirmed by pathological examination, and patients with multi-organ metastasis of lung cancer, end-stage patients, severe liver and kidney dysfunction, or cachexia were excluded from our study.

### Cell transfection

Lung cancer cell line A549 (ATCC, Manassas, VA, USA) cultured in RPMI-1640 medium with 10% fetal bovine serum, were seeded into 6-well plates, and transfected with plasmids, miR-6884-5p mimics or corresponding negative control (RiboBio Company, Guangzhou, China) for 48 h using Lipofectamine 3000 (Invitrogen, Waltham, MA, USA) according to the manufacturer’s instructions. Full length of S100A16 cDNA sequence was inserted into pcDNA3.1 to construct S100A16 plasmid for its overexpression.

### Western blot

Protein was extracted using commercial RIPA lysis buffer. Cell-extracted proteins (25 μg) were loaded into SDS-PAGE gel and then transferred to the PVDF membrane, which then was blocked with non-fat milk (5% in PBST). The membrane was further incubated with corresponding primary antibodies at 4° C for 12 h, including anti-S100A16 polyclonal antibody (1:3000), anti-MMP2 monoclonal antibody (1:2000), anti-E-cadherin monoclonal antibody (1:1000), anti-N-cadherin monoclonal antibody (1:1000), and anti-GAPDH monoclonal antibody (1:4000). All antibodies were purchased from Abcam (Shanghai, China). After that, HRP-conjugated secondary antibody was further used for incubation. The bands were visualized using an ECL substrate. GAPDH was used as the loading control.

### Luciferase assay

Briefly, A549 cells (6×10^4^ cells per well) were co-transfected with luciferase reporters containing wild-type (WT) or mutant S100A16 3’=UTR with miR-6884-5p mimics/ negative control for 48 h. After the incubation, the luciferase enzyme activity was determined using the dual-luciferase reporter assay (Promega, Madison, WI, USA).

### qRT-PCR

TRIzol reagent was employed to extract the total RNA using RNesay Mini Kit (Qiagen, Valencia, CA, USA) following the standard protocol. qRT-PCR was performed with SYBR green master mix (Thermo Fisher, Waltham, MA, USA). *GAPDH* was used as a control for mRNA, and U6 was used as a control for miRNA. The sequences of all the primers were shown listed below (5’-3’):

miR-6884-5p:

Forward AGAGGCTGAGAAGGTGATGT,

Reverse GAACATGTCTGCGTATCTC;

U6:

Forward CTCGCTTCGGCAGCACA,

Reverse AACGCTTCACGAATTTGCGT;

*S100A16:*

Forward GCTCCAGAAAGAGCTGAACCAC,

Reverse ATGCCGCCTATCAAGGTCCAGT;

*MMP2:*

Forward TACAGGATCATTGGCTACACACC,

Reverse GGTCACATCGCTCCAGACT;

*E-cadherin:*

Forward CGAGAGCTACACGTTCACGG,

Reverse GGGTGTCGAGGGAAAAATAGG;

*N-cadherin:*

Forward TCAGGCGTCTGTAGAGGCTT,

Reverse ATGCACATCCTTCGATAAGACTG;

*GAPDH:*

Forward GAGTCAACGGATTTGGTCGTATTG,

Reverse CCTGGAAGATGGTGATGGGATT.

The mRNA expression of the genes was determined using the 2^-ΔΔCT^ method.

### Statistical analysis

Violin plot was used to show patients’ data, and other data were shown as means ± SD. Two groups were analyzed using an unpaired t-test with Welch’s correction, and multiple groups were analyzed by Welch’s ANOVA test followed by Dunnett’s T3 multiple comparisons test, using GraphPad software.
